# Assessment of the correlation between clinical and radiological outcomes in patients suffering from mild to moderate cervical spine dysfunction symptoms: a prospective study

**DOI:** 10.1186/s13018-022-03455-0

**Published:** 2022-12-22

**Authors:** Xiao-ping Niu, Wei-Hua Zhu, Lu Wang, Gao-nian Zhao, Ji-tao Liu, Ai-bing Huang

**Affiliations:** 1 Postgraduate School, Dalian Medical University, Dalian, 116000 Liaoning China; 2 Department of Rehabilitation, Affiliated Hospital 5 of Nantong University (Taizhou People’s Hospital), Taizhou, 225300 Jiangsu China; 3 Department of Nursing, Affiliated Hospital 5 of Nantong University (Taizhou People’s Hospital), Taizhou, 225300 Jiangsu China; 4 Department of Orthopedics, Affiliated Hospital 5 of Nantong University (Taizhou People’s Hospital), Taizhou, 225300 Jiangsu China

**Keywords:** Cervical disc degeneration, Magnetic resonance imaging, Cervical spine dysfunction symptoms

## Abstract

**Background:**

Neck pain and cervical disc degeneration (CDD) are common findings. Valid data on correlation between clinical scores and radiological grade of CDD in patients with mild to moderate clinical disability are not available. The study has been designed to investigate the correlation between clinical and radiological outcomes in these patients.

**Methods:**

A cohort of 150 patients who suffered from mild to moderate cervical spine dysfunction symptoms from September 2020 to May 2021 was enrolled. We evaluated functional status using Japanese Orthopaedic Association scores (JOA), the visual analog scale, and the Neck Disability Index. We assessed the CDD with magnetic resonance imaging-based grading systems. We analyzed relationships between radiological grades of CDD and clinical symptoms along with demographic data.

**Results:**

One hundred thirteen patients [mean age 44.78, 78 (69%) females] were finally included. CDD occurred most at the C5–C6 level, with 56.93% of higher grade III from Miyazaki. The grades of Miyazaki (*P* < 0.05) and the scores of Nakashima (*P* < 0.05) were positively correlated with the duration of symptoms, and the severity of the CDD increased with aging (*P* < 0.01). Moreover, we correlated patients’ JOA scores with the current scoring and grading systems, especially the grades of Miyazaki (*P* < 0.01) and the scores of Nakashima (*P* < 0.01).

**Conclusion:**

Increasing grades of CDD paralleled decreasing JOA scores in the population studied.

## Introduction

Cervical disc degeneration (CDD) is a progressive condition that exists along a continuum of pathologic process, leading to neck pain, disability, and dysfunction symptoms [1, 2]. The cost of treating chronic neck pain is especially high, approximal $4261 per person per year [3].

Degeneration is associated with decreases in the proteoglycan and water in the nucleus pulposus and its demonstrated nucleus intensity, nucleus structure, distinction of nucleus and annulus, and disc height [4, 5]. Grading systems have been developed for assessing CDD, such as Pfirrmann grades [6], Miyazaki grades [7], modified Thompson grades [8, 9] and Nakashima grades [10]. Those image-based classification focuses on the imaging characteristics of the intervertebral disc, such as the signal intensity of the nucleus and annulus, the nucleus structure, and the disc height. Markotić et al. [11] applied Pfirrmann grades to evaluate CDD in 112 patients, and the results showed that a higher level of education was a risk factor leading to CDD. Also, Chen et al. [12] used the Miyazaki grades to demonstrate that cigarette smoking could accelerate the process of CDD, leading to more severe neck-shoulder pain. The relationship between MRI findings on cervical spine and factors influencing CDD has been documented extensively [13–17]. Some studies demonstrated that CDD on MRI was observed in the asymptomatic subjects [18] [1–3] [1–3] [1–3]. Matsumoto et al. [19] found that 85 (90.4%) patients demonstrated positive degenerative MRI findings. Moreover, the painless annular tears were detected by discography from the asymptomatic volunteers, which often escape MRI detection.

However, previous studies did not investigate the association between grading of CDD and clinical symptoms, and it remains unclear to what extent these changes are responsible for the clinical symptoms of the patient.

The present study was conducted to investigate the correlation of current radiological grading systems of CDD and clinical outcomes in patients with mild to moderate cervical spine dysfunction symptoms.

## Methods

### Ethical approval and subjects

This study was approved by the Medical Ethics Committee of our Hospital (Ethics Number: KY2020134) and registered in the Chinese Clinical Trial Registry (Registration Number: ChiCTR2100047228). All patients gave written informed consent. From September 2020 to May 2021, we enrolled a total of 150 patients with neck pain. Subjects who underwent cervical 3.0T MRIs with satisfactory signal-to-noise ratio were included. The exclusion criteria were as follows: history of cervical spinal surgery or trauma, age < 19 years or > 65 years, ankylosing spondylitis, imaging evidence of concurrent myelopathy, and spine fracture. After excluding 33 subjects who refused the examination of cervical 3.0T MRI, two subjects with unsatisfactory signal-to-noise ratio, and two subjects whose MRI data were not available, 113 patients were included in the final analysis. Baseline demographic data were recorded.

### Clinical symptoms assessment

The functional status was assessed by an independent physician using Japanese Orthopaedic Association (JOA) scores [20], visual analog scale (VAS) [21], and Neck Disability Index (NDI) [22, 23]. The JOA scoring system consists of seven categories: motor function of upper and lower extremity; sensory function of upper extremity, trunk, and lower extremity; and function of the bladder, which was widely used to assess the severity of neurologic function based on the scoring system; the severity of myelopathy was divided into three levels: severe (0–11points), moderate (12–14points), mild (15–17points). The VAS was used to estimate the variations in intensity of neck pain; the levels of pain intensity were classified into three groups: slight (VAS score lower than 3 points), moderate ((VAS score 4–6 points), severe (VAS score higher 7 points). The NDI was used as a measure of neck pain; there are five levels based on the scoring system: no disability (0–4 points), mild disability (5–14 points), moderate disability (15–24 points), severe disability (25–34 points), complete disability (35–50 points).

### CDD assessment

**Fig. 1:**
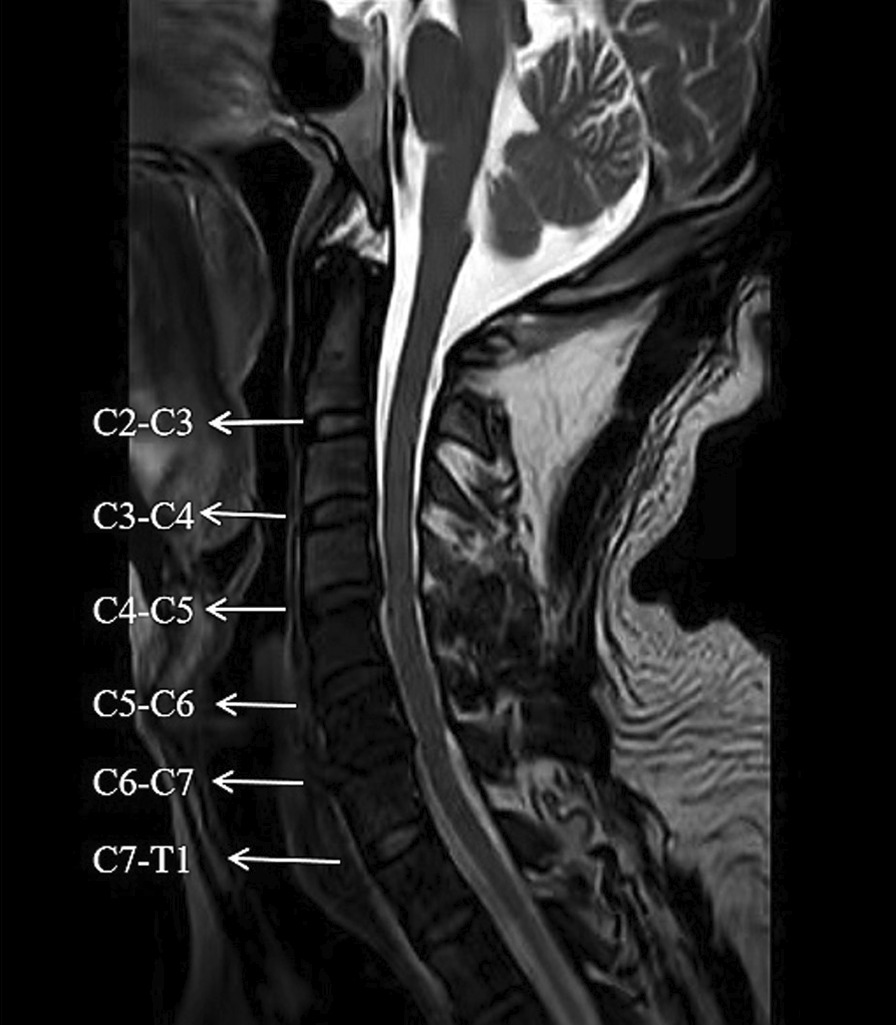
Sagittal T2‐weighted cervical MR imaging of a patient. Pfirrmann classifications of the cervical discs were as follows: C2–3 was Grade 2, C3–4 was Grade 3, C4–5 was Grade 3, C5–6 was 4, C6–7 was Grade 5, and C7–T1 was Grade 2. The total Pfirrmann scores of the patient were 19. Likewise, the total Miyazaki scores = 1 + 2 + 3 + 3 + 4 + 1 = 14, total modified Thompson scores = 0 + 1 + 2 + 1 + 3 + 0 = 7, and the total Nakashima scores = 1 + 1 + 1 + 2 + 3 + 1 = 9

We performed sagittal T1, T2‐weighted and axial T2‐weighted cervical MR imaging for each patient to evaluate disc degeneration. The grading of disc degeneration was primarily based on Pfirrmann grades [6], Miyazaki grades [7], modified Thompson grades [8, 9], and Nakashima grades [10]. Six cervical levels (C2–3, C3–4, C4–5, C5–6, C6–7, and C7–T1) were chosen, and finally 678 discs were assessed. We calculated the sum of these six levels of disc scores (Fig. 1). Modic changes (MC) and high-intensity zones (HIZ) found on MRI were also recorded. The MC were divided into three different types according to the presence of edema, fat, or sclerosis [15, 24]. Type I changes show decreased signal intensity on T1-weighted images and increased signal intensity on T2‐weighted images. Type II changes show increased signal intensity on both T1-weighted and T2‐weighted images. Type III changes show decreased signal intensity on both T1-weighted and T2‐weighted images. Location of the Modic changes in relation to endplate was recorded as entire, central, anterior, or posterior. The HIZ was defined as a bright white signal on T2‐weighted MR images, located in the posterior annulus fibrosus of the intervertebral disc [25, 26]. Also, Cobb angle was drawn on T2-weighted sagittal view MR image to evaluate the state of the cervical curves [27], which was defined as the angle between a line parallel to the base of C2 and the line parallel to the base of C7. Positive values higher than 5° were defined as cervical lordosis. Negative values below 5° indicated cervical kyphosis. The degree of Cobb angle between − 5° and + 5° was indicated cervical rectification. All the evaluation and measurement were taken in the picture archiving and communication system (PACS).

### Statistical analysis

All statistical analysis was performed with IBM SPSS version 25.0, and values were expressed as mean ± standard deviation (SD). The reliability of the measurements was assessed by examining the intraclass correlation coefficients (ICC) [28]. For intraobserver repeatability, all the measurements were repeated by the primary observer for 10 randomly selected cases 2 weeks later. The primary observer was an attending surgeon with more than 15 years of experience in spine surgery. For interobserver repeatability, all the measurements were repeated by an independent radiologist. Spearman’s test was used to determine the correlations. Correlation was regarded as weak for absolute *r* values in the range 0.2–0.39, moderate 0.4–0.69, and strong 0.7–1. A *P* value of < 0.05 was considered statistically significant [29].

## Results

A total of 113 participants were included in this study, consisting of 35 men and 78 women. Baseline demographic data and cervical clinical scores are presented in Table 1.

**Table 1:** Profiles of 113 patients

Variables	*N* (%)	(*X* ± *S*)
*Gender*		
Male	35 (31.0)	
Female	78 (69.0)	
*BMI*		24.26 ± 3.46
≤ 24 (kg/m^2^)	53 (46.9)	
> 24 (kg/m^2^)	60 (53.1)	
*Age*		44.78 ± 9.49
≤ 40 years	40 (35.4)	
> 40 years	73 (64.6)	
*Duration of symptoms*		
Less than 3 days	7 (6.2)	
Less than 3 months	42 (37.2)	
Less than 3 years	54 (47.8)	
More than 3 years	10 (8.8)	
*Cobb angle*		9.14 ± 8.07
≥ 5°	50 (44.2)	
< 5°	63 (55.8)	
*Clinical symptoms*		
*The score of JOA*		14.49 ± 1.34
Severe (0–11 points)	2 (1.77)	
Moderate (12–14 points)	46 (40.71)	
Mild (15–17 points)	65 (57.52)	
*The score of VAS*		4.90 ± 1.73
Slight(≤ 3 points)	35 (30.97)	
Moderate (4–6 points)	46 (40.71)	
Severe (≥ 7 points)	32 (28.32)	
*The score of NDI*		13.84 ± 7.27
No disability (0–4 points)	14 (12.39)	
Mild disability (5–14 points)	56 (49.56)	
Moderate disability (15–24 points)	35 (30.97)	
Severe disability (25–34 points)	8 (7.08)	
Complete disability (35–50 points)	0 (0)	

*BMI* Body mass index, *JOA* Japanese Orthopaedic Association, *VAS* visual analog scale, *NDI* Neck Disability Index, *X* ± *S* mean ± standard deviation

A total of 678 cervical discs of the 113 patients were evaluated. C5-6 were the most severely degenerated segments, and the details are summarized in Table 2. The intraobserver and interobserver ICC values for radiographic grading systems were good, respectively (0.67–0.80 and 0.71–0.83). Based on the above calculation method, the mean value of total scores was 17.84 ± 2.99 in Pfirrmann grades, 16.82 ± 3.67 in Miyazaki grades, 7.42 ± 2.57 in modified Thompson grades, and 12.04 ± 2.57 in Nakashima grades. Additionally, the percentages of Modic changes and HIZ in our cohort were 0.30%, 3.90%, respectively.

**Table 2:** Numbers and percentages of the discs distribution according to the mentioned grading system (*n* = 678)

	C2–3 (*n*, %)	C3–4 (*n*, %)	C4–5 (*n*, %)	C5–6 (*n*, %)	C6–7 (*n*, %)	C7–T1 (*n*, %)
*The grading of Pfirrmann*						
I	0 (0)	0 (0)	0 (0)	0 (0)	0 (0)	0 (0)
II	41 (6.05)	26 (3.83)	27 (3.98)	15 (2.21)	20 (2.95)	69 (10.18)
III	53 (7.82)	68 (10.03)	52 (7.67)	50 (7.37)	64 (9.44)	34 (5.01)
IV	19 (2.80)	18 (2.65)	31 (4.57)	36 (5.31)	25 (3.69)	9 (1.33)
V	0 (0)	1 (0.15)	3 (0.44)	12 (1.77)	4 (0.60)	1 (0.15)
*The grading of Miyazaki*						
I	2 (0.29)	3 (0.44)	0 (0)	1 (0.15)	11 (1.62)	22 (3.24)
II	50 (7.37)	32 (4.72)	34 (5.01)	22 (3.24)	25 (3.69)	62 (9.14)
III	51 (7.52)	57 (8.41)	49 (7.23)	43 (6.34)	44 (6.49)	22 (3.24)
IV	10 (1.47)	21 (3.10)	24 (3.54)	36 (5.31)	25 (3.69)	4 (0.59)
V	0 (0)	0 (0)	6 (0.88)	11 (1.62)	8 (1.18)	0 (0)
*The grading of modified Thompson*						
0	13 (1.92)	11 (1.62)	8 (1.18)	6 (0.88)	11 (1.62)	42 (6.19)
I	97 (14.31)	71 (10.47)	53 (7.82)	34 (5.01)	55 (8.11)	60 (8.85)
II	3 (0.44)	29 (4.28)	44 (6.49)	58 (8.55)	38 (5.60)	10 (1.47)
III	0 (0)	2 (0.29)	8 (1.18)	15 (2.21)	9 (1.33)	1 (0.15)
*The grading of Nakashima*						
I	30 (4.42)	20 (2.95)	16 (2.36)	11 (1.62)	13 (1.92)	52 (7.67)
II	73 (10.77)	76 (11.21)	72 (10.62)	53 (7.82)	66 (9.73)	68 (10.03)
III	10 (1.47)	17 (2.51)	25 (3.69)	43 (6.34)	32 (4.72)	3 (0.44)
IV	0 (0)	0 (0)	0 (0)	6 (0.88)	2 (0.29)	0 (0)
*Modic changes*						
I	0 (0)	0 (0)	0 (0)	0 (0)	0 (0)	0 (0)
II	0 (0)	1 (0.15)	0 (0)	1 (0.15)	0 (0)	0 (0)
III	0 (0)	0 (0)	0 (0)	0 (0)	0 (0)	0 (0)
*HIZ in the cervical spine*						
No	113 (16.67)	112 (16.52)	110 (16.22)	101 (14.90)	104 (15.34)	113 (16.67)
Yes	0 (0)	1 (0.15)	3 (0.44)	12 (1.77)	9 (1.33)	0 (0)

*HIZ* high-intensity zones

**Fig. 2:**
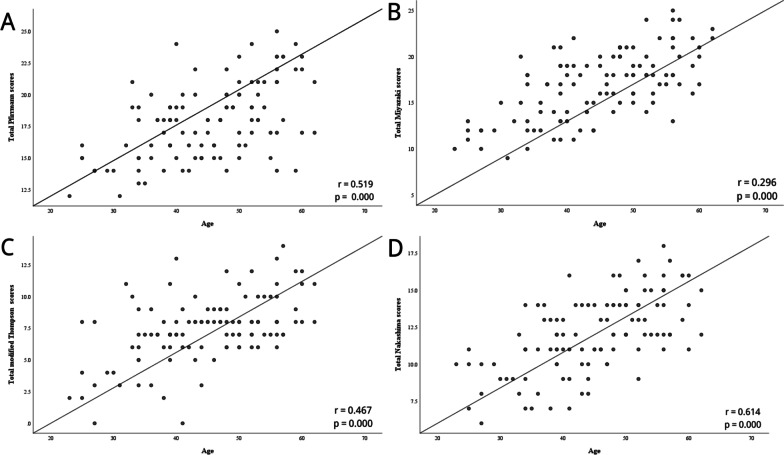
Correlation between age and the sum of these degree of cervical disc degeneration. **a** Age and total Pfirrmann scores; **b** age and total Miyazaki scores; **c** age and total modified Thompson scores; **d** age and total Nakashima scores

**Fig. 3:**
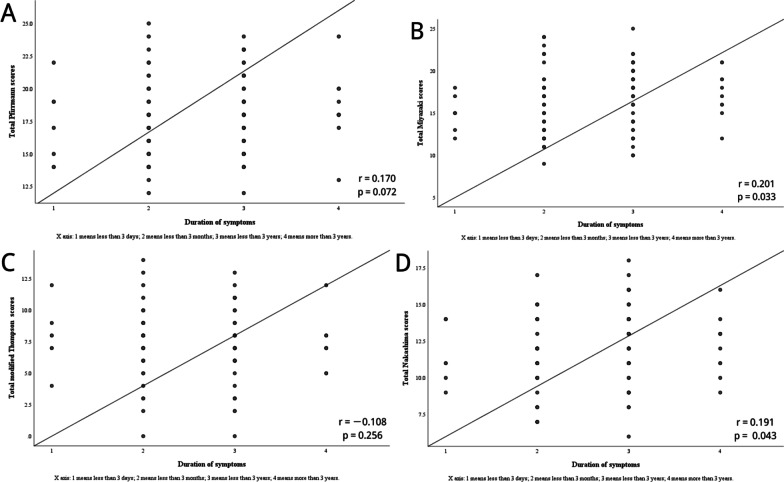
Correlation between duration of symptoms and the sum of these degree of cervical disc degeneration. **a** Duration of symptoms and total Pfirrmann scores; **b** duration of symptoms and total Miyazaki scores s; **c** duration of symptoms and total modified Thompson scores; **d** duration of symptoms and total Nakashima scores

**Fig. 4:**
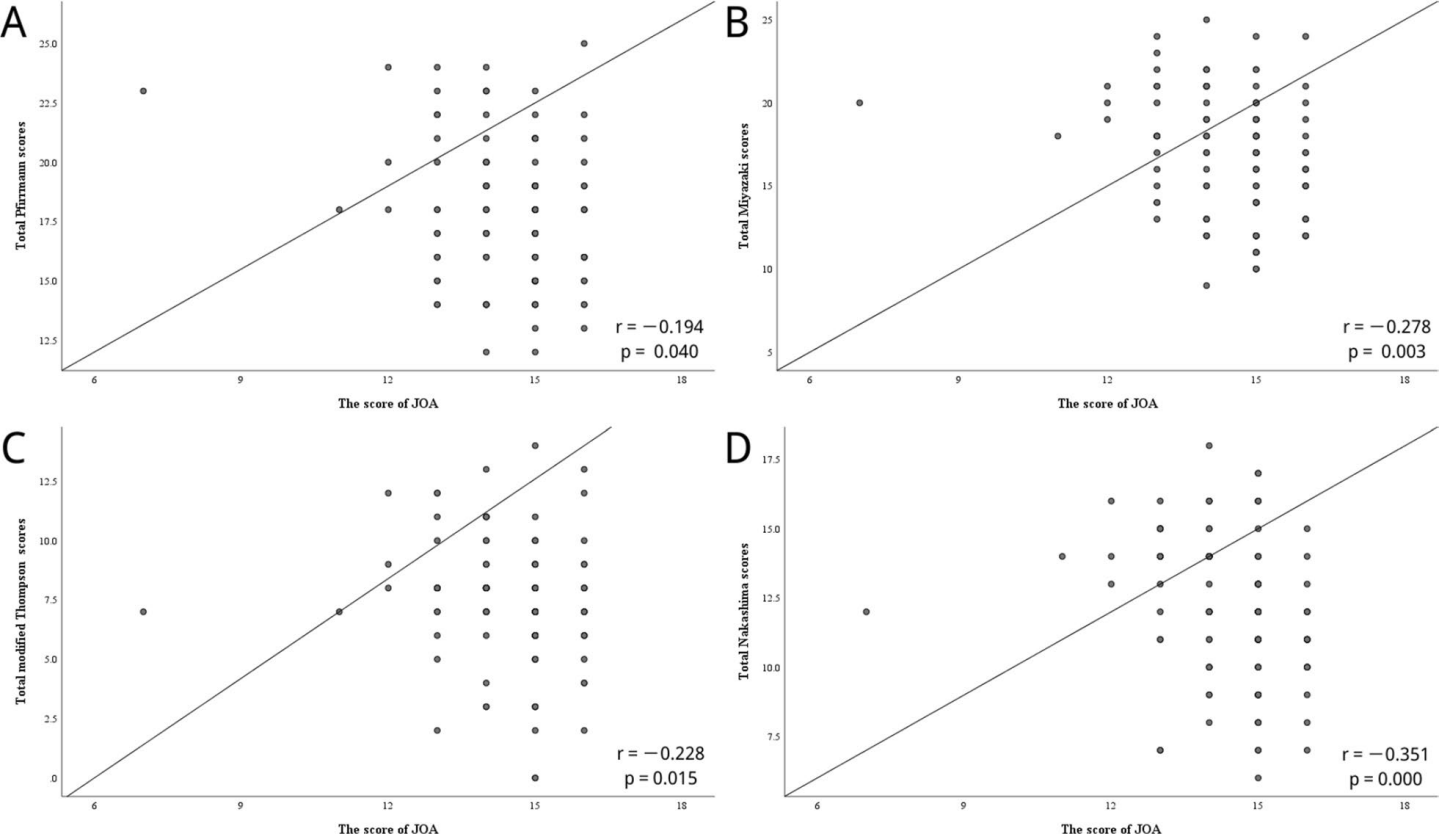
Correlation between the score of JOA and the sum of these degree of cervical disc degeneration. **a** The score of JOA and total Pfirrmann scores; **b** the score of JOA and total Miyazaki scores; **c** the score of JOA and total modified Thompson scores; **d** the score of JOA and total Nakashima scores

The relationships between radiological grading of CDD and clinical factors are summarized in Table 3. The results indicated that age correlated moderately with radiological grading systems (*r* = 0.30–0.61, *P* < 0.01) (Fig. 2). Additionally, the duration of symptoms revealed a weakly positive correlation with both total Miyazaki scores (*r* = 0.20, *P* < 0.05) and total Nakashima scores (*r* = 0.19, *P* < 0.05) (Fig. 3). Compared with the VAS and the NDI, the impairment of the JOA correlated weakly with current grading systems (Fig. 4). There was no relationship between the radiological grading of CDD and the Cobb angle (*P* > 0.05).

**Table 3:** Results of Spearman’s test (*n* = 113)

Variables	*P* value			
	Total Pfirrmann scores	Total Miyazaki scores	Total modified Thompson scores	Total Nakashima scores
Age	0.000**	0.000**	0.000**	0.000**
BMI	0.105	0.370	0.800	0.682
Gender	0.955	0.480	0.641	0.288
Duration of symptoms	0.072	0.033*	0.256	0.043*
Cobb angle	0.895	0.300	0.943	0.795
The score of JOA	0.040*	0.003**	0.015*	0.000**
The score of VAS	0.303	0.286	0.127	0.786
The score of NDI	0.587	0.256	0.421	0.348

*p < 0.05, ** *p* <0.01

*BMI* Body mass index, *JOA* Japanese Orthopaedic Association, *VAS* visual analog scale, *NDI* Neck Disability index

## Discussion

This is the first study to investigate the association of current radiological grading systems of CDD and clinical outcomes in patients with mild to moderate cervical spine dysfunction symptoms. We found that there was a significant negative correlation between the current radiographic grading systems and the JOA scores.

CDD occurs throughout life and has been measured by physical and biologic mechanisms [30, 31]. Abundance and structure of the macromolecules vary, starting during childhood, and with the process of aging the overall aggrecan and water content of the disc decrease, especially in the nucleus [30, 31]. There is a growing body of evidence showing that aging has a negative impact on the intervertebral disc [17, 32, 33]. Our study also demonstrated this relationship. In addition, the present study found that the most common level to have radiological CDD was the C5–6 level, which was similar to the results of other studies [1, 34]. Thirdly, in our study we found that the percentages of Modic changes and HIZ in all cervical spine were 0.30%, 3.9%, respectively. These results were different from the results of Tsuji et al. [15] study, who found that of Modic changes affected 47 intervertebral disc levels (3.48%) in all 1158 cervical intervertebral levels. The study conducted by Nguyen demonstrated that 58 patients (6.7%) within the 861 patients were found HIZs in their cervical spine [16]. The patients in our cohort with only mild to moderate cervical spine dysfunction symptoms were the likely cause of this difference.

Impairment of discs, muscles, and joints can cause pain in the cervical spine [35]. It has been established that MRIs provide an effective technique for showing early pathologic changes in the cervical spine. Previous study demonstrated that abnormal MR morphology of cervical spine was a more common finding in cervical pain sufferers than in asymptomatic volunteers [18]. Another study conducted by Siivola et al. [36] has shown that disc herniation was the only MRI finding significantly associated with neck pain. The authors pointed out that unusual MRI findings were common in asymptomatic and symptomatic young adults. However, the study used a fairly small sample with the age from 19 to 65, and so its findings have to be considered preliminary results. Similarly, in a 20-year prospective longitudinal study, Daimon et al. [1] also found that there was no relationship between the progression of degeneration on MRI and the development of clinical symptoms. In present study, we found that the current radiographic grading systems were associated with JOA scores. This is the first result, to our knowledge, to evaluate the relationship between clinical disability and radiographic grading systems. The findings indicated that pathophysiological changes of cervical spine verified on MRI seem to explain parts of the clinical disability in this cohort. As we can found from previous study, cervical dysfunction symptoms were related to aging [37], work-related [38], stress experienced [39], and other factors. JOA [20] offered a more sophisticated system to evaluate cervical dysfunction, which included motor function, sensory function and bladder function; it was sufficiently reliable, with a weighted kappa coefficient of more than 0.4 [40]. In another study, Nischal et al. [34] also showed that there was a strong correlation between the mJOA scores and diffusion tensor imaging matrix parameters. More studies on the current topic are needed to confirm the results.

## Limitations

The findings of this study must be seen in light of its limitations. One limitation was that the sample size was small. Therefore, a larger number of patients should have been enrolled to define the association more clearly. The second limitation was that, compared with X-rays, MRI may not gold standard for the assessment of cervical sagittal balance, which was defined as the distance between C2 plumbline and the posterior upper corner of C7 [30]. However, in this study, Cobb angles were drawn on MRI; thus, the generalizability of the results was limited, which Cobb angles had no relationship between cervical disc degeneration.

## Conclusion

Focusing on CDD, this study has analyzed the correlation of current radiographic grading systems and clinical outcomes in patients with mild to moderate cervical spine dysfunction symptoms. Increased grading of in CDD paralleled decreased JOA scores in the population under study.

## Data Availability

The datasets used and analyzed in this study are available from the corresponding author on reasonable request.

## Abbreviations

CDD: Cervical disc degeneration
JOA: Japanese Orthopaedic Association scores
VAS: Visual analog scale
NDI: Neck Disability Index
